# Palmer’s Dental Depot

**Published:** 1856-12

**Authors:** 


### PALMER'S DENTAL DEPOT.

Mr. Palmer keeps a fine assortment of dental instruments of the best quality at eastern prices. He is also the Cincinnati agent for Orum & Armstrong's teeth. These teeth are rapidly gaining in popularity, but not more rapidly than they deserve. See advertisement.

